# Latent Tuberculosis: Two Centuries of Confusion

**DOI:** 10.1164/rccm.202011-4239PP

**Published:** 2021-03-30

**Authors:** Marcel A. Behr, Eva Kaufmann, Jacalyn Duffin, Paul H. Edelstein, Lalita Ramakrishnan

**Affiliations:** ^1^ Department of Medicine,; ^2^ McGill International Tuberculosis Centre, and; ^3^ Meakins-Christie Laboratories, McGill University, Montreal, Quebec, Canada;; ^4^ Hannah Professor Emerita of the History of Medicine, Queen’s University, Kingston, Ontario, Canada;; ^5^ Department of Pathology and Laboratory Medicine, Perelman School of Medicine, University of Pennsylvania, Philadelphia, Pennsylvania; and; ^6^ Molecular Immunity Unit MRC, Laboratory of Molecular Biology and Cambridge Institute of Therapeutic Immunology and Infectious Diseases, Department of Medicine, University of Cambridge, Cambridge, United Kingdom

The current dogma is that ∼2 billion individuals, making up a quarter of the world’s population, are latently infected with *Mycobacterium tuberculosis* (1–4). It is thought that latent tuberculosis infection (LTBI), defined by the presence of immunoreactivity to tuberculosis antigens in the absence of clinical and radiologic manifestations of tuberculosis (TB) disease, can reactivate even decades after infection to cause transmissible disease. Therefore, the high prevalence of LTBI is seen as a critical barrier to global TB eradication. Yet our recent analyses of studies spanning 5 decades find that the majority of TB-immunoreactive individuals have cleared their infection while retaining immunological memory of it (5, 6). Three lines of evidence suggest that this is the case (5–7): *1*) Longitudinal studies show that of the minority of infected individuals who progress to disease, most do so within months to 2 years; *2*) TB immunoreactivity can persist after curative TB treatment; *3*) The majority of TB-immunoreactive individuals have cleared infection as evidenced by their failure to get TB disease even after profound immunosuppression. Based on these findings, it appears that the number of people harboring live *M. tuberculosis* is substantially lower than previously thought (8). Work published over the last several decades indicates that many TB experts have come to similar conclusions as our recent analyses (5, 6). How then did latent TB infection come to be seen as a lifetime sentence?

## Latent TB Terminology: Origins, Evolution, and Pervasion/Pervasiveness

Since 1971, the American Thoracic Society (ATS) has regularly issued TB treatment guidelines (9). In 1994, these guidelines issued recommendations for the treatment of TB disease and TB infection with no mention of the word “latent” (10–13). In 1999, the ATS issued for the first time a separate guideline that specifically addressed “latent tuberculosis infection” (14) Not only did the terminology for TB infection change to LTBI, the terminology for its treatment also changed—from “preventive treatment” to “treatment of LTBI.” The recommended treatment for LTBI (isoniazid monotherapy) remained unchanged from that for its namesake (TB infection) in 1994. The 1999 guidelines offered neither an explanation for the new name nor a definition (14). Rather, the definition was in a separate document published contemporaneously that contained guidelines for the diagnosis of TB. Here, LTBI was defined as “a positive reaction to the tuberculin skin test (TST), negative bacteriologic studies (if done), and no clinical, bacteriologic or radiologic evidence of active tuberculosis” (15). In 2017, the updated guidelines modified the LTBI definition to “a state of persistent immune response to stimulation by *Mycobacterium tuberculosis* antigens with no evidence of clinically manifest active TB.” (16).

**Figure 1.**
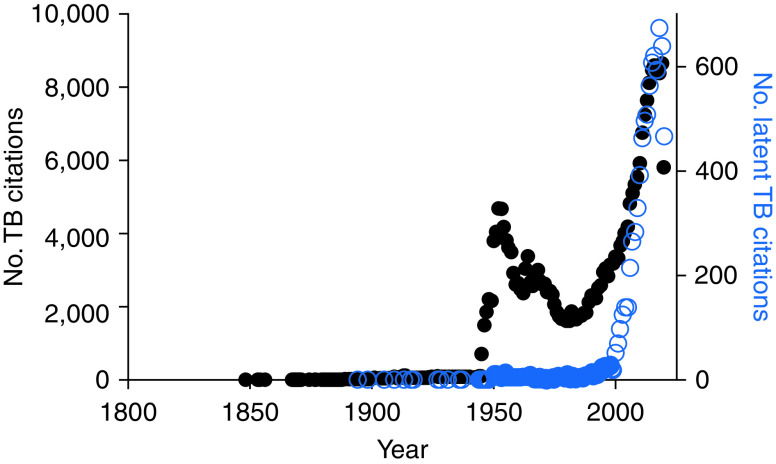
Publications on TB (left *y*-axis) and latent TB (right *y*-axis). TB = tuberculosis.

The influential role of the ATS guidelines is reflected by endorsements from key organizations, such as the Centers for Disease Control and Prevention, the Infectious Diseases Society of America, and the American Academy of Pediatrics (15). It is not surprising then that their changed terminology for the exact same clinical condition—radiological evidence of inactive TB and/or TB immunoreactivity—had a profound influence on the field. Indeed, a PubMed search suggests this, showing a surge of papers using the term “latent tuberculosis” starting in 2000 (Figure 1). We did, however, find occasional indexed papers on latent TB as far back as 1894. Surely, the definition of latent TB in the 19th and early 20th century papers could not have been the same as the current one, given that the earliest articles predated tuberculin testing. What could latent TB have meant in the pretuberculin era? To understand this, we took our search back further, and identified the term “phthisie latent” (latent phthisis) in papers as early as 1819 (17). Thus, the concept of latent TB predated not only tuberculin testing but also, by more than 60 years, the discovery of the tubercle bacillus.

## 19th Century: Latent TB Is a Postmortem Diagnosis Referring to a Host with Tuberculous Pathology in the Absence of Symptoms

The characteristic gross pathology of TB in people dying of the disease was known since 1680 (18, 19). In the early 19th century when the role of anatomopathology in medicine expanded, French and German physicians described pathological changes of TB in necropsied lungs of individuals who had not had symptomatic TB before they died, and they coined the term “latent” TB or phthisis (as TB was called then) to describe this situation. Pierre-Charles-Alexandre Louis, devoting 39 pages of his 1825 treatise to the subject, inferred that despite the lack of a compatible clinical course, the latent phthisis found on autopsy must have been the cause of death (20) (for the original French, *see* the online supplement). His contemporary, the renowned French physician René Laennec (inventor of the stethoscope), came to an altogether different conclusion. In the first edition of his seminal contribution “De l’ausculation mediate” (1819) (17) (for the original French, *see* the online supplement), Laennec first described tubercles in patients who died of an acute illness (i.e., not TB) “in whom the phthisis had always been latent” and expanded on the latent theme and his conclusion in the second edition of his work 7 years later (21). Observing numerous miliary tubercles in individuals who had had no clinical signs of TB, he inferred that the majority of patients with phthisis are asymptomatic with the latent lesions being an incidental finding. Until Laennec, TB had been thought to be uniformly fatal. It was Laennec who proposed the novel idea that TB lesions could regress spontaneously (22).

The finding of TB lesions on autopsy of asymptomatic individuals dying of other causes was repeatedly validated over the next decades in France and Germany (23–25). These physicians recognized the implications of their findings. In 1833, German physicians concluded that there were two forms of the lung disease, acute and latent, and regarded the latent form as presymptomatic TB, as evidenced by their statement that “the latent form, having been hidden for a shorter or longer period of time, could only be recognized after greater progression” (23) (for the original German, *see* the online supplement). In 1868, the French physician Jean Antoine Villemin (who had demonstrated that TB is an infectious disease in 1865, 17 years before Koch did) wrote that tubercles can exist in certain subjects without any appreciable signs of disease (25, 26) (for the original French, *see* the online supplement). In 1882, the German physician Paul Clemens von Baumgarten wrote in “Uber latente Tuberkulose” that the latent state had been described as the time after the transmission of the TB poison until the outbreak of the disease (27) (for the original German, *see* the online supplement)—effectively an incubation period *avant la lettre.* In sum, the 6 decades preceding Koch’s determination of the bacterial cause of TB saw the growing awareness of the presence of TB lesions in individuals dying of other causes than TB and the nearly universal conclusion that these represented either presymptomatic TB or asymptomatic TB that would not have progressed.

Koch’s demonstration of culturing *M. tuberculosis* in 1882 and importantly the establishment of animal models of TB disease—rabbits by Villemin and guinea pigs by Koch—set the stage for the addition of a microbiology component to the pathology studies of the previous decades (25, 26, 28). In 1890, Alfred Loomis reported on his search for live tubercle bacilli in 48 patients who had died suddenly, without a prolonged illness (i.e., not from TB) (29). He was able to recover tubercle bacilli from rabbits or guinea pigs inoculated with the lymph nodes of eight patients, leading him to conclude that tubercles could be deposited in bronchial glands. Of 19 patients in whom the nodes had nonspecific changes, such as enlarged or pigmented, 7 revealed tubercle bacilli on animal inoculation; in contrast, they were detected in only 1 of 11 patients with normal appearing lymph nodes. Introducing his findings, Loomis referred to “the presence of tubercle bacilli in the swollen lymphatic glands of children who gave no evidence whatever of tuberculosis in the lungs or other parts of the body,” stating that “in most cases of phthisis in children the disease has been preceded by a long-existent, but latent, glandular tuberculosis.” The addition of a microbiologic assay was a major advance in understanding the natural history of TB. A 1900 paper by Francis Baup summarizes this nicely: “It was only in 1894 that M. Lermoyez, having examined adenoid vegetations, which were considered suspicious, found, in midst of adenoid tissue, tubercles and Koch’s bacilli and demonstrated thus in the clearest possible way the possibility of a concealed, latent infection of the tonsil by the bacillus of Koch” (30). It is important to note that the addition of the microbiologic component to the pathologic findings of latent TB did not cause a change in the definition of latent TB. It remained a postmortem diagnosis, referring to the host who harbored gross pathology and TB bacteria in the absence of symptoms. It was inferred to reflect tuberculous pathology developing in the incubation period of those who were in the presymptomatic phase of infection when they died of other causes or, alternatively, serendipitously identified transient tuberculous pathology that would have resolved spontaneously had the individual not died of other causes.

## 20th Century: Latent TB Remains a Postmortem Diagnosis but Now Referring to the Tubercle Bacilli Recovered from Autopsy Tissue without TB Pathology

In the early 20th century, investigators continued to look for the anatomic sites of TB infections in individuals without overt TB who had died from other causes, now with an increased microbiological focus. This included inoculating susceptible animals with lymph nodes that did not have classic tuberculous pathology. Because tubercle bacilli were occasionally recovered from such lymph nodes, a new line of thinking emerged that bestowed on TB bacteria a special attribute—the ability to reside in tissues without producing pathology. However, these normal tissues that had obviously been used as “negative controls” were in reality composed of two distinct, often conflated samples: *1*) abnormal tissue without the specific manifestations pointing to tuberculosis and *2*) completely normal tissue. Nevertheless, in a complete shift of the meaning, latent TB now referred to these bacteria rather than to the asymptomatic host in whom bacteria had been found.

Multiple papers illustrate the conflation of both normal and nonspecifically abnormal tissues as the source of the tubercle bacilli and the shifted definitions. In a 95-page paper in 1905, Francis Harbitz, working in Norway, describing “investigations strictly beyond the scope of my research and … made somewhat desultorily,” reported that “In my series of 142 autopsies . . . latent tubercle bacilli were demonstrated in 18 of the 91 cases in which there was no gross nor histological sign of tuberculosis” (31). Under the heading of normal nodes, he wrote: “the occurrence in lymph nodes of tubercle bacilli demonstrable by inoculation, without concurrent macroscopic or microscopic changes” (31). Yet, later in the text, he stated that normal nodes did not contain tubercle bacilli. Referring to the search for “latent tubercle bacilli in apparently normal tissue,” he wrote: “I have also examined for tubercle bacilli sections from nodes adjacent to those from which successful inoculations were made . . . I made such controls in 10 cases, with negative result.”

Also in 1905, Anton Weichselbaum and Julius Bartel, in “Zur Frage der Latenz der Tuberkulose,” (On the question of latency in tuberculosis) reported on eight autopsies in Germany (32). They wrote that inoculation of a small number of lymph nodes apparently free of tuberculous alteration produced disease in guinea pigs. Their work too was internally contradictory. At one point they described guinea pigs that displayed tubercles at necropsy having been inoculated with lymph nodes that had been negative for TB (by microscopic inspection for TB), yet they noted that in some cases the lymphatic tissue was swollen, and in some cases reddened (for the original German, *see* the online supplement). Reports of the presence of tuberculosis in normal appearing tissue rapidly influenced the veterinary TB literature as well. Henri Vallée in his 1909 paper “Occult tuberculosis” issued the following warning: “The attention of veterinary surgeons, and more particularly of meat inspectors, should be drawn, however, to the fact that in animals, as in man, the invasion of gland tissue by Koch’s bacillus, under natural conditions even more than in experimental tuberculosis, does not always lead to the rapid production of macroscopic lesions, this temporary condition of infection by the bacilli being capable of existing for a variable and sometimes a very long time. To this particular condition of gland infection some authors, principally in Germany, applied the term ‘latent tuberculosis’” (33).

Chung Yik Wang of Edinburgh was the first to explicitly acknowledge the switch in the use of latent to describe the bacillus rather than the host. In his 1916 paper “An Experimental Study of Latent Tuberculosis” published in *The Lancet*, he distinguished two potential meanings of latent TB: clinical and bacteriological. “Latent tuberculosis, in a sense other than that applied clinically, may be defined as an infection of the body with tubercle bacilli without the infection showing a specific development—that is a new formation or change of tissue which can be recognised by macroscopic or microscopic examination” (34). He then provided findings from the postmortem examination of 32 individuals who were found free of signs of tuberculosis at necropsy. Of these, 48 samples from 29 individuals failed to reveal tubercle bacilli following inoculation of guinea pigs. In the remaining three individuals, each had one positive lymph node and all three were described as “slightly enlarged,” “swollen, congested,” and “soft, congested.” Therefore, Wang’s findings (TB bacilli with nonspecific pathology) again contradicted his assertion that TB bacteria can be present in tissues without pathology.

Nevertheless, through these papers in the early part of the 20th century, a microbiological definition of latent TB emerged that referred to tubercle bacilli isolated from tissue without tubercular pathology. From these findings, the notion took hold that tubercle bacilli could become avirulent within the host and thereby reside in tissue without causing pathology. This concept is exemplified in Harbitz’s paper: “We have the demonstration in a comparatively large number of cases, of the presence of latent tubercle bacilli in the lymph nodes, and especially in the cervical nodes. The following questions then arise: Do these bacilli possess their usual virulence? How long may the bacilli be supposed to have lain latent in the lymph node”? (31). Likewise, Weichselbaum and Bartel proposed the possibility that the tubercle bacilli that have penetrated an organ do not multiply for a certain time and therefore do not produce any changes (32) (for the original German, *see* the online supplement). Yet, from the start, both the definition and the concept were flawed as the studies that reported recovering the latent bacteria frequently did so from the tissue that *was* abnormal, albeit without the characteristics of established TB. Weichselbaum and Bartel further stated that the tubercle bacilli may be carried off into other organs where they produce a manifest TB while the bacilli in the first organ do not multiply, that is, remain latent. The idea that there were dormant bacteria that lay low and do not replicate was reiterated in a 1933 paper from Harold Eugene Robertson: “a tuberculous infection may remain latent or dormant for many years” and “these organisms might remain latent without multiplication” (35). Yet, again, Robertson’s assertion was not supported by his data. He presented only histopathologic findings without microbiologic investigations.

An alternative explanation, largely absent from these papers, was the idea proposed by Vallée: that these were recently arrived bacteria that had simply not had the time to provoke full-blown tuberculous pathology (33). If true, the bacteria were in the process of inducing tuberculous pathology, as indicated by the nonspecific inflammatory pathology seen in many of the lymph nodes from which they were recovered. Furthermore, not all investigators accepted the new bacteria-centric definition. For instance, Eugene Opie referred to a host-centric process, writing in 1927: “Latent tuberculosis may be defined as tuberculous infection which is unaccompanied by significant symptoms evident to the patient or by physical signs discovered by the physician” (36). Despite the paucity of data regarding latent bacilli, the new definition of latent TB to describe avirulent or dormant bacteria capable of long-term residence in the host without causing pathological changes stuck for the rest of the century (37–46).

## 21st Century: Latent TB Refers to a Host Who Is TB Immunoreactive in the Absence of TB Disease

It is on this historical backdrop that at the tail end of the 20th century, latent TB acquired yet another new definition (15). This 21st-century definition of latent TB has circled back to the host (Table 1). However, in contrast to the 19th-century postmortem pathological diagnosis, it is now an antemortem immunological diagnosis. This new LTBI diagnosis requires a pertinent positive—TB immunoreactivity—and a pertinent negative—the absence of clinical manifestations. Each of these presents diagnostic quandaries.

**Table 1.** Definitions of Latent Tuberculosis over Two Centuries

Year	Author	Positive Finding	Pertinent Negative	Latent Modifies
1825–1826	Louis, Laennec	Tubercles	Signs and symptoms	Host
1905	Harbitz	Culture	Pathology	Bacterium
1999	ATS guidelines	Immunoreactivity	Signs and symptoms	Host

*Definition of abbreviation*: ATS = American Thoracic Society.

The TB immunoreactivity tests (TST and, more recently, IFN-γ release assays, or IGRAs) are neither 100% specific nor sensitive. A positive TST/IGRA cannot distinguish present infection from present disease, nor can it distinguish present from past infection. Several lines of evidence suggest that the majority of individuals thought to have LTBI have actually cleared their TB infection (5, 8, 36). Conversely, a negative TST/IGRA test is observed in 10–40% of HIV-negative individuals with culture-confirmed pulmonary TB, and an even higher percentage of patients with miliary TB (47–49). Moreover, a new concept has emerged that recognizes people who have epidemiologic evidence of exposure without demonstrable T-cell–mediated immune responses to mycobacterial antigens, and such individuals would test negative (50, 51). For these “resisters,” there is evidence through alternative assays that infection has occurred despite the subject being TST/IGRA negative (52). In sum, the immunologic definition of infection overlooks people who are infected but do not manifest classical immunoreactivity yet includes people with immunoreactivity who have already cleared their infection (Table 2).

**Table 2.** Tuberculous Infection versus Immunoreactivity

	Live *M. tuberculosis* Present = Tuberculous Infection		No Live *M. tuberculosis* Present
TB immunoreactive	TB	Tuberculous infection, no disease*^†^	Cleared*^‡^
TB nonimmunoreactive	TB	Tuberculous infection, no disease^†^	Never infected^§^

*Definition of abbreviations*: *M. tuberculosis* = *Mycobacterium tuberculosis*; TB = tuberculosis.

* People who are currently offered preventive TB treatment.

^†^ People who may benefit from preventive TB treatment.

^‡^ Or nonspecific TB immunoreactivity.

^§^ Or infected and cleared without ever developing immunoreactivity.

Regarding the pertinent negative—the absence of clinical manifestations—this term lends itself to different interpretations by clinicians and microbiologists. If clinical manifestations mean signs and symptoms, then individuals with subclinical TB (bacteriologically positive but negative on symptom screening) could be considered to have latent TB (53–55). Indeed, it is likely that some of the patients described by Laennec and Louis would have been sputum culture positive, had the test existed 2 centuries ago. If absence of clinical manifestations refers to a negative sputum culture, as suggested by others (56), TB is unique among bacterial infectious diseases in that the state of infection is diagnosed with a negative test for the microbe.

## Shifting Definitions Have Converged into Confusion with Clinical, Research, and Public Health Implications

**Figure 2.**
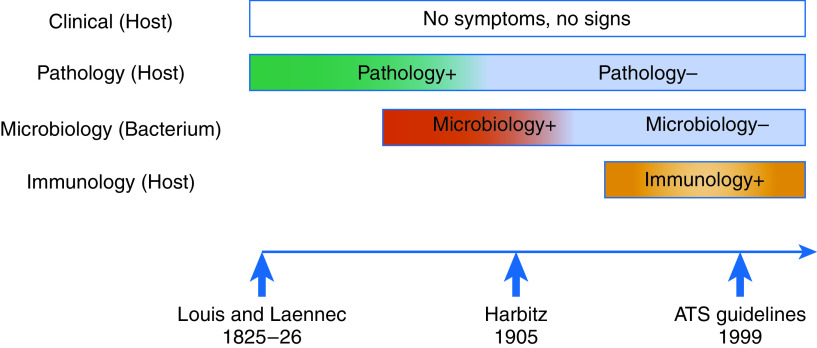
Shifting definitions of latent tuberculosis over time. Both pathology and microbiology positivity were ascertained postmortem. Immunologic testing is done antemortem. ATS = American Thoracic Society.

**Figure 3.**
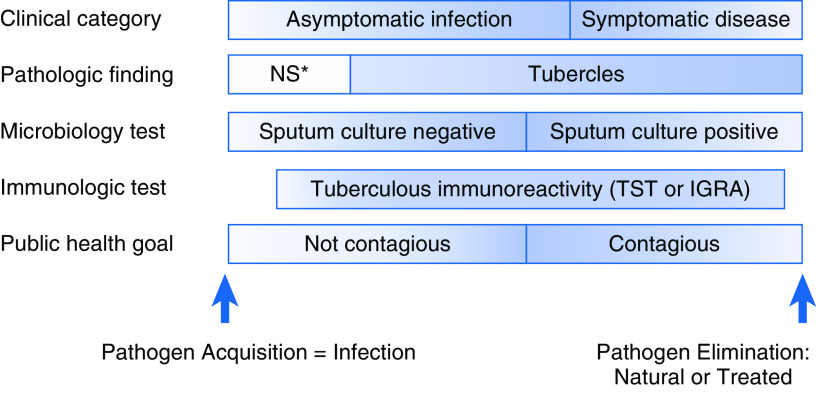
*Mycobacterium tuberculosis* infection from different vantages. Tuberculous reactivity starts later than infection because of the time to mount a measurable immune response and is presented as earlier to become negative to represent waning responses with severe infection. *NS denotes nonspecific. IGRA = IFN-γ release assay; TST = tuberculin skin test.

Languages evolve, as do the meanings of individual words, so it is not surprising that latent TB has meant different things since 1819 (Figure 2). The problem is that the old definitions cannot be subsumed into the new ones if the meaning has changed—the different definitions refer to different concepts. Recognizing that the currently used term LTBI encompasses a test result and a conceptual process, we propose that it be disambiguated into the two. For the former, the patient manifests tuberculous immunoreactivity and may or may not be still infected. For the latter, the more scientifically accurate term is “*M. tuberculosis* infection” or “tuberculous infection,” the supraset comprising symptomatic and asymptomatic infection, as well as contagious and noncontagious individuals (Figure 3).

The distinction between tuberculous immunoreactivity and tuberculous infection offers advantages in multiple spheres of TB treatment, research, and public health. Under the current definition of LTBI, immunoreactive people who have cleared the bacteria meet the definition of “infection.” Meanwhile, people who fail to meet the immunological definition of “infection” include patients with culture-confirmed TB, as well as individuals with noncanonical immune responses. Recognizing the imperfection of current tests allows us to look beyond these assays, through translational research, to identify better biomarkers of tuberculous infection. Such markers have the potential to prioritize patients for preventive therapy and spare uninfected people treatments that cannot benefit them. Currently, candidate biomarkers are being evaluated in TST/IGRA-based categories, such that an apparently poor test performance may simply reflect the imperfect comparator (57). An accurate categorization of who has current versus past infection is likely to be valuable for clinical research as well. Trials of vaccine candidates aim to prevent disease, or infection in people deemed to be uninfected. Biomarkers of infection could present an efficacy signal sooner than disease-based outcomes and could potentially be detected with smaller study sizes. For such studies, we need suitable assays of who is truly infected, to select uninfected subjects before exposure, to ascertain the endpoints of the intervention, and to learn from the exposed subjects who naturally clear their infection. Similarly, assays for who is truly infected could benefit antibiotic trials looking at prevention of disease (58). Finally, from a public health standpoint, it is important to have accurate global estimates of who is really infected, to understand the specific challenges in different settings and distribute resources accordingly. To make progress in these many areas, we need to start with a clear and consistent definition of tuberculous infection (Box 1).

**Box 1. Proposed Nomenclature for Tuberculous Infection**

**Uninfected:** no infection, no disease, may or may not be TB immunoreactive.

**Tuberculous infection (TBI):** infected with live *Mycobacterium tuberculosis*.

**Tuberculous infection no disease (TBInd):** tuberculous infection, asymptomatic and culture negative, may or may not be TB immunoreactive.

**Tuberculosis (TB):** symptomatic and/or culture positive, may or may not be TB immunoreactive.
